# Admission levels of serum biomarkers have additive and cumulative prognostic value in traumatic brain injury

**DOI:** 10.1038/s41598-024-64125-1

**Published:** 2024-06-19

**Authors:** Ida A. Kaaber, Maj Lesbo, Thea O. Wichmann, Dorte Aa. Olsen, Mikkel M. Rasmussen, Ole Brink, Lars C. Borris, Claus V. B. Hviid

**Affiliations:** 1 Department of Clinical Biochemistry, Aarhus University Hospital, 8200 Aarhus, Denmark; 2 Department of Clinical Medicine, Aarhus University, Aarhus, Denmark; 3 Department of Ortopedic Surgery, Viborg Regional Hospital, Viborg, Denmark; 4 Department of Neurosurgery, Aarhus University Hospital, Aarhus, Denmark; 5 Department of Biochemistry and Immunology, Lillebaelt Hospital, University Hospital of Southern Denmark, Vejle, Denmark; 6 Department of Ortopedic Surgery, Aarhus University Hospital, Aarhus, Denmark; 7 Department of Clinical Medicine, Aalborg University, Aalborg, Denmark; 8 Department of Clinical Biochemistry, Aalborg University Hospital, Aalborg, Denmark

**Keywords:** Biomarkers, Epidemiology, Outcomes research, Brain injuries, Medical research, Biomarkers, Predictive markers, Prognostic markers

## Abstract

Elevated levels of CNS-derived serum proteins are associated with poor outcome in traumatic brain injury (TBI), but the value of adding acute serum biomarker levels to common clinical outcome predictors lacks evaluation. We analyzed admission serum samples for Total-Tau (T-Tau), Neurofilament light chain (Nfl), Glial fibrillary acidic protein (GFAP), and Ubiquitin C-terminal hydrolase L1 (UCHL1) in a cohort of 396 trauma patients including 240 patients with TBI. We assessed the independent association of biomarkers with 1-year mortality and 6–12 months Glasgow Outcome Scale Extended (GOSE) score, as well as the additive and cumulative value of biomarkers on Glasgow Coma Scale (GCS) and Marshall Score for outcome prediction. Nfl and T-Tau levels were independently associated with outcome (OR: Nfl = 1.65, p = 0.01; T-Tau = 1.99, p < 0.01). Nfl or T-Tau improved outcome prediction by GCS (Wald Chi, Nfl = 6.8–8.8, p < 0.01; T-Tau 7.2–11.3, p < 0.01) and the Marshall score (Wald Chi, Nfl = 16.2–17.5, p < 0.01; T-Tau 8.7–12.4, p < 0.01). Adding T-Tau atop Nfl further improved outcome prediction in majority of tested models (Wald Chi range 3.8–9.4, p ≤ 0.05). Our data suggest that acute levels of serum biomarkers are independently associated with outcome after TBI and add outcome predictive value to commonly used clinical scores.

## Introduction

Traumatic brain injury (TBI) is a leading cause of mortality and disability among adults and is estimated to strike 69 million globally each year^1,2^. It is a highly heterogenous disease and prognostication of outcome can be challenging. Prediction of outcome is based on clinical assessment, clinical scores, and neuroradiology^3^. In effort to improve outcome prediction, more complex statistical models including the International Mission for Prognosis and Analysis of Clinical Trials in TBI (IMPACT) model have been developed^4^. However, such models are not routinely adapted to clinical use maybe because they are too complex to integrate into clinical workflow. This leaves tremendous potential for improvement of outcome prediction if more applicable paraclinical measures are developed.

Along this line, an immense research effort has been put into development of serum biomarkers for TBI as easily accessible and objective tools for assessment of prognosis^5–8^. Central nervous system (CNS)-derived proteins are studied as candidate biomarkers as they are released into the peripheral blood upon damage to neuronal tissue^9^. With recent technical advances, these proteins are now detectable in serum, making them accessible with a simple blood test^10,11^.

Total-Tau (T-Tau), Neurofilament light chain (Nfl), Glial fibrillary acidic protein (GFAP), and Ubiquitin C-terminal hydrolase L1 (UCHL1) are candidate biomarkers of TBI that have been intensively investigated. T-Tau is a microtubule regulatory protein primarily found in cortical unmyelinated axons, Nfl is a cytoskeletal protein abundantly expressed in myelinated axons, GFAP is an astrocyte-specific cytoskeletal protein, and UHCL1 is a neuron-specific enzyme involved in the production of ubiquitin^12–15^. As such, each biomarker provides information on injury to distinct cell populations, and levels of these serum biomarkers have been associated with lesions on CT head scans^6,16,17^, TBI severity^16,18,19^, functional outcome^6,18^, and mortality following TBI^6,20^, supporting their potential as a clinical tool to improve assessment of injury severity and outcome prediction.

However, previous studies are often done on homogenous cohorts of TBI patients with isolated head injuries. This is far from the clinical setting where TBI mostly co-occurs with other injuries that could impact biomarker performance^9^. Furthermore, serum biomarkers are often investigated as isolated predictive factors rather than exploring their additive value to existing clinical outcome predictors. Lastly, combining biomarkers might further improve outcome prediction, as a panel of biomarkers with different cellular origins can provide more comprehensive information of brain injury. In this study, we evaluate serum biomarker performance, sampled on hospital admission, in a cohort of unselected, traumatized patients with and without TBI. Furthermore, we explore the potential additive value of serum biomarkers to existing clinical outcome scores and lastly; if the addition of a combination of serum biomarkers further improves outcome prediction.

## Methods

### Study population

This study was conducted using biobank material from the established *Systematic Urine Evaluation for Activation of Coagulation in Severe Trauma* (SURVIVE) cohort^21^. The cohort consists of 418 trauma patients admitted to the level I trauma center of Aarhus University Hospital, Denmark from March 2017 to March 2018. In the SURVIVE study, all admitted trauma patients were evaluated for inclusion. Exclusion criteria in the SURVIVE study were: (1) patients younger than 18 years; (2) patients dead upon arrival; (3) pregnancy; (4) patients not fulfilling the Advanced Trauma Life Support (ATLS) criteria for trauma team activation^22^; and (5) patients who declined or withdrew consent. For this study, an additional 10 patients were excluded because of unsure identification due to missing civil registration number.

In the present study, trauma patients were classified as TBI victims based on retrospective review of medical records. TBI was defined as report of head trauma or other potential causes of brain injury which comprises all injuries to the head described in medical records, including superficial lacerations, abrasions, and contusions to the face and scalp. Patients admitted with attempted strangulation or other means resulting in cerebral hypoxia, as well as patients with intracranial hemorrhage of uncertain cause were also included in the TBI group (accounts for two patients). Patients with traumatic spinal cord injury with/without concomitant TBI were excluded from the present study (n = 12). The classification of patients were performed by the first author and verified by an experienced senior consultant in neurosurgery.

### Ethics approval and informed consent

The study was conducted in accordance with the Helsinki declaration and has been approved by the Scientific Ethics Committee for the Central Denmark Region (1-10-72-205-16 and 1-10-72-204-16) and the Danish Data Protection Authority (1-16-02-452-16). All patients or their next of kin provided written informed consent to participate in the study. If patients were incapable of providing consent, this was obtained from next of kin as well as a physician not involved in patient treatment or the conduction of the study. If patients gained ability to provide consent within 72 h of admission, consent was asked to be confirmed or denied^21^.

### Data collection

In the SURVIVE cohort all patient data was collected retrospectively from the local trauma registry and medical records. The local trauma registry contains information on all patients admitted to the trauma center of Aarhus University Hospital^23^. Information on patient’s time of injury, injury type, prehospital treatment interventions, information on vital signs (heart rate, respiratory frequency, and blood pressure) and Glasgow Coma Score (GCS) registered upon admission, as well as the abbreviated injury severity (AIS) score was extracted. The New Injury Severity Score (NISS)^24^ was subsequently calculated on the basis of the AIS. In 31 patients, GCS was missing in the trauma registry and the GCS was therefore retrieved from medical records as registered at the time of admission. Information on prehospital parameters contained in the registry is extracted from prehospital medical records and from the trauma chart by a team of junior doctors with special training^25^.

An electronic medical record covering all clinical and paraclinical patient information is kept at all Danish hospitals. In the SURVIVE study, records were used to collect information on time of hospital admission, time of blood sampling, age, sex, and pre-existing antiplatelet and anticoagulant treatment. If information on anti-platelet/-coagulant therapy was missing, patients were registered as not receiving this treatment. Information on blood transfusions and performance of surgical procedures within 72 h of admission was collected. A detailed description of the collection and classification process has been published previously^21^.

For the purpose of the present study, the pupil reflex score (PRS) was retrieved from medical records of the initial 24 h of hospital stay for patients with GCS ≤ 14. Patients with unilaterally or bilaterally unresponsive pupils were registered as non-responding and patients with bilateral response as responding. The lowest PRS within 24 h of admission was registered. Patients with a GCS of 15 were registered as responding.

For the present study, the Marshall Computed Tomography (CT) Classification score was retrospectively registered based on the initial trauma CT scan. The Marshall score is a classification system consisting of six injury classes based on presence of midline shift, basal cistern compression, or hemorrhagic lesions^26^. Patients with no visible pathology was given a score of 1. CT scans were evaluated by a junior doctor with special experience in neurosurgery and verified by a senior consultant in neurosurgery.

For this study, we also retrieved TBI patient outcome measures included 1-year mortality and 6–12-month functional outcome determined by Glasgow Outcome Scale Extended (GOSE) score^27^. The GOSE consists of eight outcome categories: (1) Death; (2) Vegetative state; (3) Lower severe disability; (4) Upper severe disability; (5) Lower moderate disability; (6) Upper moderate disability; (7) Lower good recovery; (8) Upper good recovery. We based the scores on data from medical records regarding patient consciousness, independence in everyday life, and ability to work and engage in social activities. Upper good recovery (GOSE = 8) represents full return to life as before TBI. For the present study, the GOSE score was dichotomized into a favorable (GOSE ≥ 5) and unfavorable (GOSE ≤ 4) outcome group.

An outcome predictive model approximating the IMPACT (International Mission for Prognosis and Analysis of Clinical Trials in TBI) core model was constructed for TBI patients in this study^4^. This outcome model consists of the PRS, admission GCS, and age. It differs from the original IMPACT model by use of total GCS score as opposed to GCS motor score. The IMPACT prognostic model was developed by Steyerberg et al. for predicting 6-month outcome in adult patients with moderate to severe TBI (admission GCS ≤ 12)^4^.

### Blood samples

Blood samples were collected upon arrival, 15 (± 3) hours, and 72 (± 6) hours after admission. Patients discharged or transferred to another hospital within 72 h of admission left the study. Thirty-two patients consented to the use of the admission blood sample only and refrained from further blood sampling, while the remaining patients provided consent to all three blood samples^21^.

Blood samples were collected from the arterial line or by venous puncture and drawn into either EDTA-, heparin- or serum tubes (BD Vacutainer®, Becton, Dickinson and Company, Franklin Lakes, NJ, USA). Samples were transported to the laboratory at room temperature and processed within one hour. Samples for chemistry-, and hematologic analysis were analyzed in our accredited (DS/EN ISO15189) clinical laboratory according to standard operating procedures. Samples for chemistry analysis (heparin anti-coagulated) were centrifuged at 3000*g*, 5 min at 22–24 °C before analysis whereas hematologic analysis (EDTA anti-coagulated) were performed using whole blood. Blood samples for serum biomarker analysis (serum samples) were allowed to clot for 30 min at 22–24 °C before being centrifuged at 3000*g*, 25 min at 22–24 °C and frozen at − 80 °C pending biochemical analysis.

### Biochemical analysis

Chemistry (plasma glucose and creatinine) and hematologic parameters (hemoglobin and hematocrit) were analyzed at the Department of Clinical Biochemistry, Aarhus University Hospital, using validated assays for routine clinical use. Chemistry parameters were analyzed using Roche Cobas 6000, while hematology parameters were analyzed using Sysmex XE-5000.

Analysis of serum biomarkers was done at the Department of Clinical Biochemistry, Aarhus University Hospital, and the Department of Biochemistry and Immunology, Lillebaelt Hospital between September and November 2022. Biomarker levels were analyzed using the Neurology 4-plex assay B kit (Quanterix Corp, MA USA) on a single molecule array (Simoa) HD-1 Analyzer (Quanterix Corp) according to the manufacturer’s instructions. All analyses were performed by a certified laboratory technician experienced in Simoa analysis. In brief, serum samples were thawed at room temperature and batched analyzed in singles in random order. A total of twelve runs were performed using two different reagent lots. A low and high control sample was included in each batch. Standard dilution was four-fold. Any samples yielding a signal over quantification or calibrator range were diluted (a maximum of 1:100) and reassayed. The technical specifications of this analysis, including the lower limit of quantification (LLOQ), the LOD, the calibrator range, and the intermediate precision expressed by the Coefficient of Variability (CV) of a high and low control can be found in Supplementary Table S1.”. One T-Tau result was half the LoD and ten UCHL1 results were below the LoD (reported range 0.2–1.6 pg/ml). These results were included in the analysis using the reported values. All NfL and GFAP results were above the LoD.

### Statistical analysis

Distribution of data was evaluated by visual inspection of inverse QQ-plots. Data are presented as absolute numbers (n) and percentages, means with standard deviation (SD) or 95% confidence intervals (CI), or as medians with interquartile range (IQR) as appropriate. Correlations between biomarkers were analyzed by Spearman’s rho (ρ). Categorical data was analyzed with Chi-squared test. Continuous data was compared by Mann–Whitney U-test or Kruskal–Wallis test followed by Dunn’s test.

To adjust the comparison of biomarker levels between TBI patients and controls, multiple linear regression analysis was performed. For each biomarker, a model was fitted with the biomarker level expressed as dependent variable and TBI status as independent variable. The analysis was adjusted for age, sex and NISS.

To investigate the effect of increasing clinical TBI severity on biomarker levels in the TBI group, multiple linear regression analysis was used. For each biomarker, a model was fitted with the biomarker level expressed as dependent variable, and measures of TBI severity (admission GCS, positive lesion on head CT, and Marshall Classification score) as independent variables. Admission GCS was categorized as mild GCS 14–15, moderate GCS 9–13, or severe GCS 3–8. Positive lesion on head CT was dichotomized and Marshall Classification score was aggregated into three groups (I, II–IV, V–VI). Analyses were adjusted for age, sex, and NISS. Biomarker levels had a screwed distribution and were ln-transformed before the regression analysis. All results were backtransformed to original scale before presentation. All regression models were checked for standard assumptions.

Ability of biomarkers to separate patients with TBI by dying/surviving and functional outcome was explored by receiver operating characteristics (ROC) curve analysis. The area under the ROC curves was compared as described by Delong et al.^28^. To ensure the completeness of data in the outcome analysis, missing admission samples of serum biomarkers were replaced by 15- or 72-h samples if available (maximum of 27–29 missing admission samples per biomarker). The effect of this approach was limited as evaluated by its impact on “time from injury to first sample”, “average admission level of the biomarkers” and the “correlation between sampling time and biomarker level” (Supplementary Table S2). Predictive value of the serum biomarkers for 1-year mortality and 6–12 months functional outcome (GOSE ≤ 4 vs. ≥ 5) after TBI was explored by logistic regression modelling. For each biomarker, four individual logistic regression models were fitted expressing mortality/GOSE as dependent variable and the biomarker as independent variable. The four models were adjusted for 1: NISS and age; 2: admission GCS and age; 3: Marshall Classification score and age; as well as 4: pupil reflex, admission GCS, and age. Biomarkers independently associated with outcome were further tested for their ability to improve established clinical outcome predictors using nested logistic regression analysis. In an initial approach, biomarkers were included individually in three logistic regression models along with; 1: Admission GCS and age; 2: Marshall Classification score and age; and 3: pupil reflex, admission GCS, and age. Subsequently, biomarkers that were weakly correlated (ρ < 0.40), were included in the same nested regression model to explore potential cumulative predictive value to clinical outcome models. The potential improvement of the nested models was evaluated by Wald Chi-Squared test. All analyses were performed using STATA version 18.0 and results were considered significant at an alpha level of 0.05.

## Results

### Study cohort characteristics

**Figure 1:**
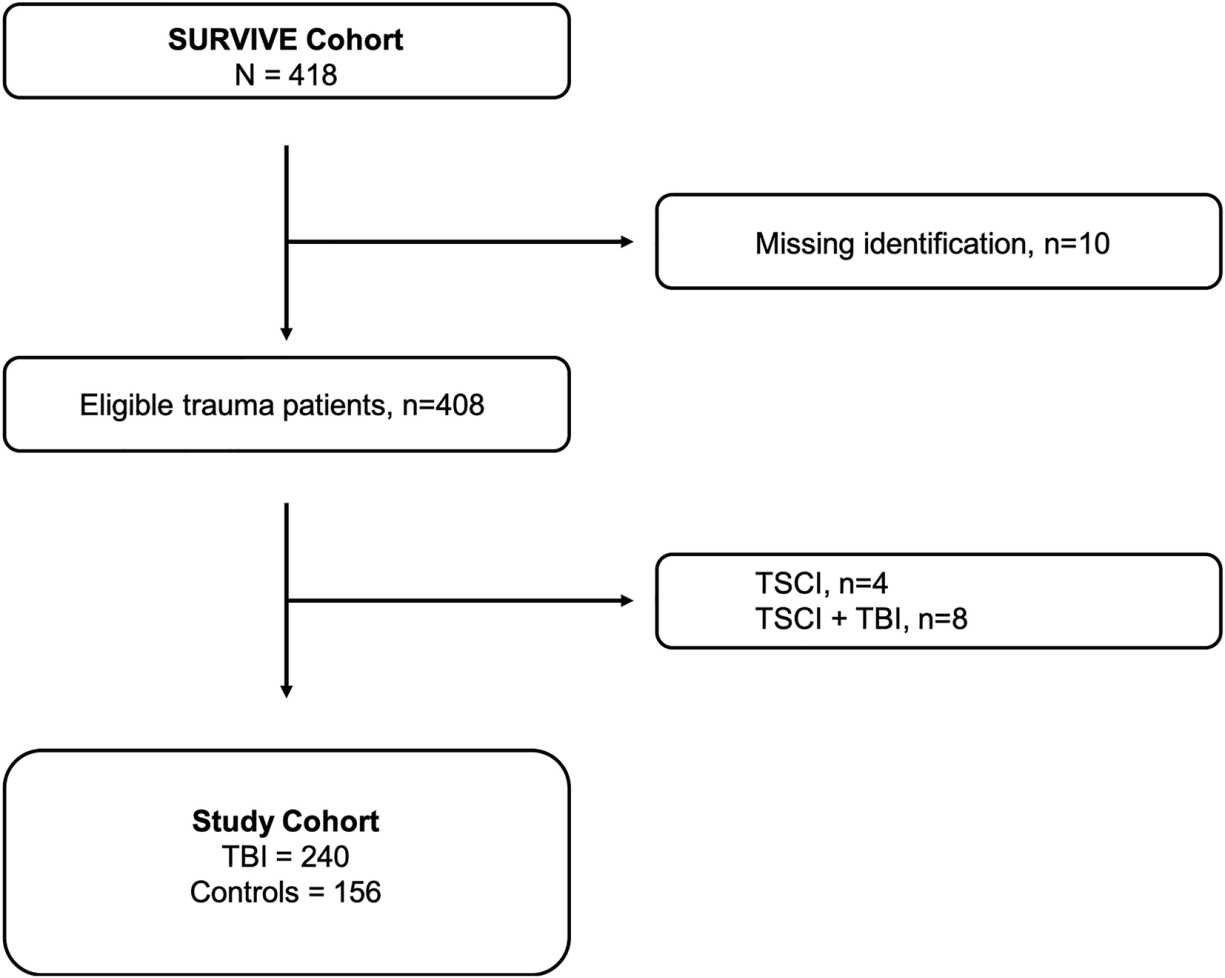
Study cohort selection process. *TCSI* traumatic spinal cord injury, *TBI* traumatic brain injury.

The patient selection process is shown in Fig. 1. Of the 396 trauma patients available for this study, 240 had suffered TBI and the remaining 156 served as controls.

The characteristics of trauma patients, treatment course, and serum biomarkers levels are presented in Table 1. Males dominated the cohort. The TBI patients were older than the controls, and more patients with TBI were in circulatory shock (shock index ≥ 0.8) and profoundly injured (NISS > 24) on admission compared with controls. One percent of controls (n = 2) had an admission GCS < 14, while this occurred in 29% of the TBI cases. Controls with GCS < 14 were due to alcohol intoxication. Admission hematocrit and creatinine levels were within normal ranges in both groups with no difference observed.

**Table 1:** Study cohort characteristics and levels of serum biomarkers on admission, after 15, and 72 h.

Characteristics	Controls (n = 156)	TBI (n = 240)	p value
Male/female	101/55 (65/35)	160/80 (67/33)	0.69
Age, median (IQR)	42 (26–54)	46 (30–66)	< 0.01
Preadmission medical treatment and biochemistry			
Anticoagulant prescription, yes/no	7/28 (20/80)	16/50 (24/76)	0.47
Platelet inhibitor prescription, yes/no	6/24 (20/80)	20/44 (31/69)	0.13
Hematocrit, median (IQR)	0.42 (0.39–0.45)	0.41 (0.39–0.44)	0.67
Creatinine, median (IQR)	79 (67–88)	77 (67–90)	0.69
Clinical severity on admission			
Time to hospital arrival, minutes, median (IQR)	54 (37–73)	55 (37–78)	0.96
Intubated on admission, yes/no	3/150 (2/96)	56/181 (24/76)	< 0.001
Shock index, > 0.8/ ≤ 0.8	9/121 (7/93)	31/175 (15/85)	0.03
Pupil reflex, yes/no	156/0	226/14	< 0.01
Glasgow coma scale (GCS)			
14–15	154 (99)	166 (69)	< 0.001
9–13	2 (1)	28 (12)	< 0.001
3–8	0 (0)	45 (19)	< 0.001
New injury severity score (NISS)			
0–8	73 (47)	103 (44)	0.45
9–15	41 (26)	40 (17)	0.02
16–24	22 (14)	41 (17)	0.43
> 24	15 (10)	51 (22)	< 0.01
Mechanism of injury			
Fall	33 (21)	76 (32)	0.02
Traffic accidents	43 (27)	89 (38)	0.05
Violence or suicide	12 (8)	18 (8)	0.94
Other	65 (42)	54 (23)	< 0.001
Surgical procedure within 72h, yes/no	55/101 (35/65)	85/155 (35/65)	0.97
Blood transfusion within 72h, yes/no	12/144 (8/92)	22/218 (9/91)	0.61
Serum biomarker levels, pg/mL, median (IQR)			
On admission			
Tau, n = 353	5.8 (2.6–13.5)	6.9 (3.3–16.9)	0.05
Nfl, n = 353	8.2 (5.8–13.9)	11.1 (6.6–24.9)	< 0.001
GFAP, n = 353	93.7 (72.7–185.4)	439.0 (155.6–1678.2)	< 0.001
UCHL1, n = 345	42.7 (19.2–106.5)	76.3 (36.1–163.7)	< 0.001
After 15 h			
Tau, n = 250	3.0 (1.6–5.1)	3.9 (2.2–8.1)	< 0.01
Nfl, n = 250	11.8 (7.2–20.9)	25.8 (13.0–56.4)	< 0.001
GFAP, n = 250	155.05 (93.1–550.9)	4328.2 (743.8–12,410.9)	< 0.001
UCHL1, n = 228	30.4 (16.7–53.3)	52.8 (26.3–131.3)	< 0.001
After 72 h			
Tau, n = 123	1.5 (0.9–3.4)	2.9 (1.3–5.9)	< 0.01
Nfl, n = 124	22.2 (13.4–39.2)	52.8 (28.1–116.5)	< 0.001
GFAP, n = 124	103.1 (61.4–157.4)	2613.9 (353.1–10,426.0)	< 0.001
UCHL1, n = 112	13.2 (8.3–20.5)	34.6 (16.3–71.2)	< 0.001

All values are presented as n (%) unless stated otherwise. Information on preadmission anticoagulant and platelet inhibitor prescriptions was only obtained from a minority of the study cohort.

*IQR* interquartile range, *GCS* Glasgow coma scale, *NISS* new injury severity score, *Nfl* neurofilament light, *GFAP* glial fibrillary acidic protein, *UCHL1* ubiquitin carboxy-terminal hydrolase L1.

### Serum biomarkers are elevated in patients with TBI

Patients with TBI had significantly higher levels of serum T-Tau, Nfl, GFAP, and UCHL1 than controls at all investigated time-points (Table 1). This finding was further explored in a linear regression model adjusted for age, sex, and NISS (Supplementary Table S3). All biomarkers were associated with TBI status at all the investigated time points in the crude analysis [minimum increase by a factor 1.44 (Tau) to maximum increase of a factor 15.72 (GFAP)]. These associations were maintained in the adjusted analysis for all biomarkers but T-Tau [minimum adjusted increase by a factor 1.16 (NfL) to maxiumu increase of a factor 11.24 (GFAP)]. Generally, the association between biomarker levels with TBI status became increasingly stronger at 15- and 72 h post-admission for all biomarkers [minimum increase observed for T-Tau (1.16–1.50–1.69 on admission, 15- and 72 h) and maximum increase observed for GFAP (3.32–9.78–11.24 on admission, 15- and 72 h)] (Supplementary Table S3)).

### Serum biomarker levels are associated with TBI severity

The association between clinical TBI severity and admission serum biomarker levels was explored by multiple linear regression (Table 2). Admission levels of all four biomarkers were positively associated with decreasing GCS, presence of lesion on head CT, as well as increasing Marshall Classification Score in the crude analysis. After adjustment for age, sex, and NISS, the association with decreasing admission GCS was maintained only for Nfl. Similarly, only GFAP levels were found to be significantly associated with presence of lesion on head CT after adjustments, while the association with increasing Marshall Classification score remained for both Nfl and GFAP in adjusted analyses. Biomarker levels in the subset of TBI patients with reduced pupil reflex was also explored (Supplementary Table S4). Reductions in pupil reflex was associated with profound elevations nearly all tested biomarkers at all investigated time points (only GFAP at 15 h and UCHL1 at 72 h did not reach significance).

**Table 2:** Association between admission levels of serum biomarkers and TBI severity.

	Tau		Nfl		GFAP		UCHL1	
	Crude	Adjusted	Crude	Adjusted	Crude	Adjusted	Crude	Adjusted
GCS	1.64 (1.30–2.07), p < 0.001	1.05 (0.86–1.30), p = 0.29	1.58 (1.32–1.89), p < 0.001	1.24 (1.09–1.41), p < 0.01	2.20 (1.63–2.97), p < 0.001	1.27 (0.99–1.64), p = 0.06	1.63 (1.29–2.06), p < 0.001	1.12 (0.90–1.39), p = 0.33
Lesion on head CT	2.24 (1.52–3.32), p < 0.001	0.70 (0.47–1.03), p = 0.07	2.27 (1.69–3.05), p < 0.001	1.22 (0.94–1.57), p = 0.13	11.45 (7.52–17.44), p < 0.001	4.44 (2.87–6.89), p < 0.001	2.32 (1.57–3.44), p < 0.001	0.81 (0.53–1.23), p = 0.31
Marshall classification score	2.08 (1.58–2.73), p < 0.001	0.82 (0.61–1.11), p = 0.20	1.90 (1.54–2.34), p < 0.001	1.29 (1.07–1.57), p < 0.01	6.56 (4.92–8.75), p < 0.001	3.57 (2.57–4.96), p < 0.001	1.96 (1.48–2.59), p < 0.001	0.84 (0.61–1.16), p = 0.28

Results of multiple linear regression analyses of the association between admission level of serum biomarkers and TBI severity. Individual models were fitted with categorized admission GCS (14–15, 9–13, 3–8), presence of lesion on head CT (yes/no), and Marshall Classification score (I, II–IV, V–VI) as independent variables and adjustments for age, sex, and NISS. The coefficients were back-transformed to the original scale and are presented with 95% confidence intervals. Number of observations in each biomarker model, crude/adjusted: Tau, Nfl, GFAP: n = 221/216; UCHL1: n = 220/215.

*TBI* traumatic brain injury, *GCS* Glasgow Coma Scale, *CT* computed tomography, *NISS* new injury severity score, *Nfl* neurofilament light, *GFAP* glial fibrillary acidic protein, *UCHL1* ubiquitin carboxy-terminal hydrolase L1.

### Serum biomarkers predict outcome after TBI

**Figure 2:**
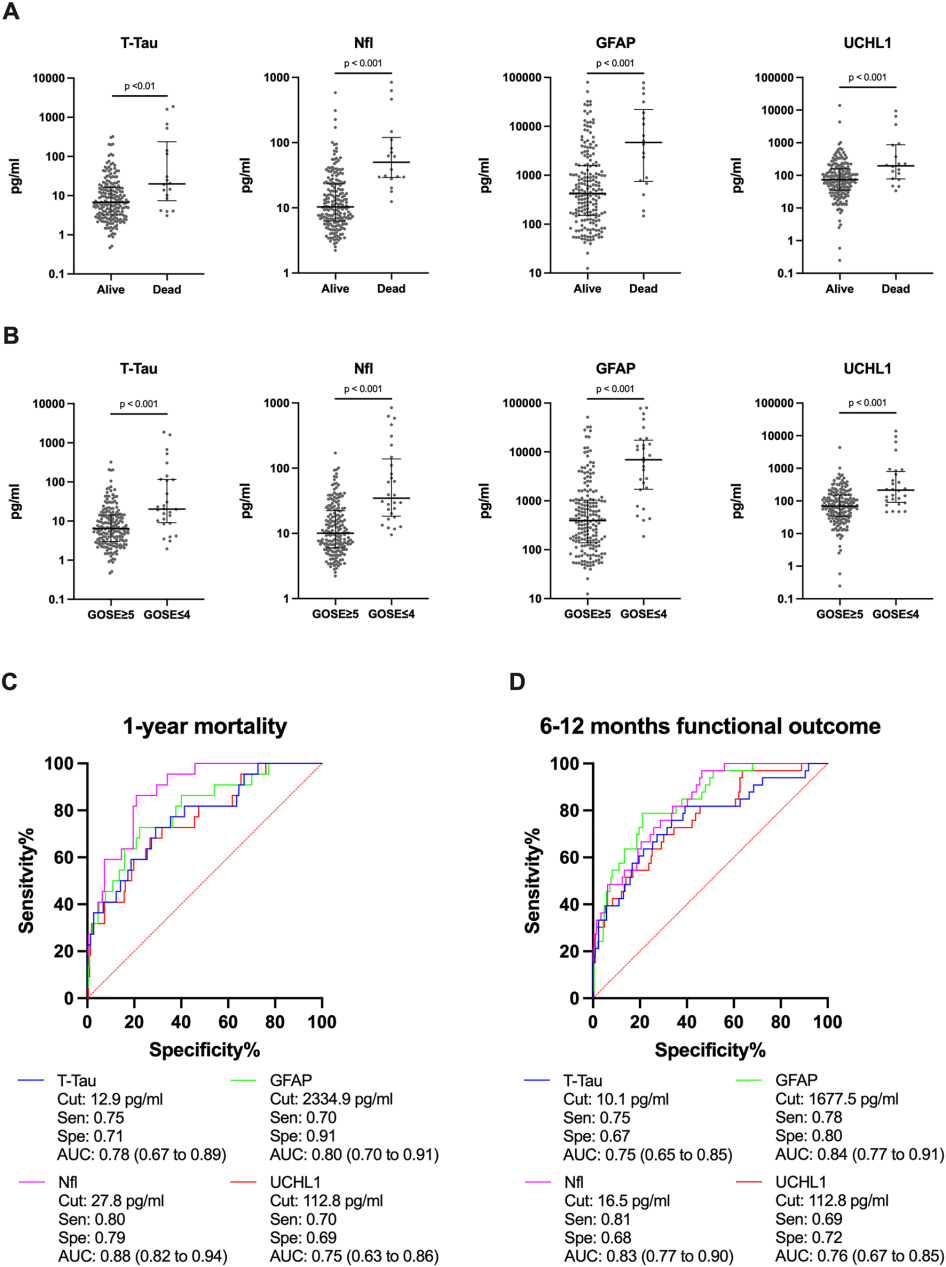
(**A**) Serum biomarker levels on admission in patients surviving/dying 1 year after TBI. Number of samples in each group: Alive, n = 203; Dead, n = 18. Biomarker levels presented with different ordinate scales. (**B**) Serum biomarker concentrations at admission in TBI patients with favorable (GOSE ≥ 5) vs. unfavorable outcome (GOSE ≤ 4). Number of samples in each group: Favorable outcome, n = 191; unfavorable outcome, n = 28. Biomarker levels presented with different ordinate scales. (**C**,**D**) ROC curves for the ability of serum biomarkers to separate patients with TBI by (**C**) 1-year mortality and (**D**) 6–12 months functional outcome (GOSE ≤ 4 vs GOSE ≥ 5). AUC with 95% CI shown along with sensitivity and specificity at defined cut points. *Nfl* neurofilament light, *GFAP* glial fibrillary acidic protein, *UCHL1* ubiquitin carboxy-terminal hydrolase L1, *GOSE* Glasgow outcome scale extended, *ROC* receiver operating characteristics, *Cut* cut point, *Sen* sensitivity, *Spe* specificity, *AUC* area under the curve.

Of the 240 patients with TBI, 21 died within 1 year of admission, and 32 had an unfavorable outcome (GOSE ≤ 4) 6–12 months after TBI. Median levels of all four biomarkers were elevated in TBI patients dying compared with TBI patients surviving head trauma (Fig. 2A). Similarly, TBI patients with an unfavorable outcome had higher median levels of all four biomarkers compared to TBI patients with a favorable outcome (Fig. 2B).

The admission levels of serum biomarkers separated the TBI outcome groups with moderate to high AUCs (Fig. 2C,D). Nfl provided the highest AUC for separating TBI patients surviving/dying, while UCHL1 provided the lowest AUC for this separation. Comparing all biomarkers, the AUC of Nfl was statistically significantly higher than the AUC of UCHL1. No other statistically significant differences in AUCs were found (Supplementary Table S5). Separating TBI patients by functional outcome, GFAP and Nfl provided the highest AUCs, while T-Tau provided the lowest. When comparing all biomarkers, no significant differences in AUCs were found for this separation (Supplementary Table S5).

The association between admission levels of serum biomarkers, with 1-year mortality and 6–12 months functional outcome was further analyzed by logistic regression modelling (Table 3). In crude analysis, all four biomarkers were significantly associated with both outcomes, but the association was most pronounced for T-Tau and Nfl, whereas GFAP and UCHL1 were less effectful. This was further explored in four individual logistic models controlling for NISS and age; GCS and age; Marshall score and age; and pupil reflex, admission GCS and age (Table 3). Nfl and T-Tau were independently associated with mortality and functional outcome in all adjusted models, except one model for prediction of functional outcome. For GFAP and UCHL1, the independent association with outcome was maintained in only 50% of adjusted models.

**Table 3:** Association between admission levels of serum biomarkers and 1-year mortality and 6–12 months unfavorable outcome in TBI patients.

Serum biomarker	Crude	Model 1	Model 2	Model 3	Model 4
1-year mortality					
Tau (per 100 pg/ml)	2.12 (1.30–3.45) p < 0.01	1.99 (1.16–3.39), p = 0.01	2.93 (1.57–5.48), p < 0.01	2.71 (1.56–4.72), p < 0.001	2.57 (1.28–5.16), p < 0.01
Nfl (per 100 pg/ml)	2.12 (1.43–3.14) p < 0.001	1.66 (1.09–2.53), p = 0.02	1.73 (1.20–2.48), p < 0.01	1.66 (1.15–2.36), p < 0.01	1.65 (1.12–2.43), p = 0.01
GFAP (per 10,000 pg/ml)	1.42 (1.16–1.75) p < 0.01	1.28 (1.02–1.60), p = 0.03	1.26 (1.06–1.51), p = 0.01	1.19 (0.97–1.46), p = 0.10	1.21 (1.00–1.45), p = 0.05
UCHL (per 100 pg/ml)	1.03 (1.01–1.06) p < 0.05	1.02 (0.99–1.05), p = 0.10	1.03 (1.01–1.06), p = 0.01	1.03 (1.00–1.05), p = 0.04	1.02 (0.99–1.05), p = 0.10
6–12 months unfavorable outcome					
Tau (per 100 pg/ml)	2.43 (1.38–4.30) p < 0.01	1.42 (0.89–2.26), p = 0.14	2.27 (1.24–4.13), p < 0.01	2.26 (1.31–3.89), p < 0.01	2.42 (1.17–5.00), p = 0.02
Nfl (per 100 pg/ml)	3.81 (1.75–8.32) p < 0.01	1.65 (0.93–2.91), p = 0.09	2.35 (1.23–4.47), p < 0.01	1.97 (1.46–4.12), p = 0.01	2.25 (1.18–4.31), p = 0.01
GFAP (per 10,000 pg/ml)	1.54 (1.20–1.97) p < 0.01	1.12 (0.91–1.36), p = 0.28	1.26 (1.03–1.55), p = 0.03	1.14 (0.92–1.41), p = 0.22	1.19 (0.96–1.46), p = 0.11
UCHL (per 100 pg/ml)1	1.12 (1.02–1.22) p < 0.05	1.06 (0.99–1.13), p = 0.10	1.09 (1.01–1.18), p = 0.03	1.09 (1.00–1.19), p = 0.05	1.07 (0.98–1.17), p = 0.11

Results of logistic regression analyses of the association between admission levels of serum biomarkers and 1-year mortality and 6–12 months unfavorable outcome. Odds ratios (OR) with 95% confidence intervals presented. Model adjustments: Model 1: NISS, age; Model 2: GCS on admission, age; Model 3: Marshall Classification Score, age; Model 4: Pupil reflex, admission GCS, and age.

*NISS* new injury severity score, *GCS* Glasgow coma scale, *Nfl* neurofilament light, *GFAP* fibrillary acidic protein, *UCHL1* ubiquitin carboxyl-terminal hydrolase L1.

### Nfl and T-Tau have additive and cumulative value for outcome prediction in patients with TBI

The association between admission levels of serum biomarkers revealed a weak correlation between Nfl and T-Tau (ρ = 0.33, p < 0.001) while the remaining biomarkers were more closely correlated (ρ = 0.40–0.62) (Supplementary Table S6). The ability of Nfl and T-Tau to improve established clinical outcome predictors was evaluated by nested logistic regression analysis (Table 4). Including Nfl or T-Tau to predictive models with GCS and age, Marshall and age, or pupil reflex, GCS and age significantly improved prediction of 1-year mortality and 6–12 months functional outcome. Furthermore, inclusion of T-Tau atop Nfl in these models improved the predictive value over Nfl alone for 1-year mortality and 6–12 months functional, except in the model with pupil reflex, GCS and age for prediction of functional outcome.

**Table 4:** Wald Chi-Squared Test for added value of Tau and Nfl individually and in combination in nested regression models for prediction of 1-year mortality and 6–12 months unfavorable outcome in patients with TBI.

				1-year mortality	6–12 months unfavorable outcome
Nested model 1					
GCS				25.2, p < 0.001	39.7, p < 0.001
GCS	Age			9.6, p < 0.01	11.2, p < 0.001
GCS	Age	Tau		11.3, p < 0.001	7.2, p < 0.01
GCS	Age	Nfl		8.8, p < 0.01	6.8, p < 0.01
GCS	Age	Nfl	Tau	8.3, p < 0.01	3.8, p = 0.05
Nested model 2					
Marshall classification score				23.5, p < 0.001	34.8, p < 0.001
Marshall classification score	Age			6.7, p < 0.01	8.0, p < 0.01
Marshall classification score	Age	Tau		12.4, p < 0.001	8.7, p < 0.01
Marshall classification score	Age	Nfl		17.5, p < 0.01	16.2, p = 0.01
Marshall classification score	Age	Nfl	Tau	9.4, p < 0.01	4.5, p = 0.03
Nested model 3					
Pupil reflex, GCS, and age				34.6, p < 0.001	21.3, p < 0.001
Pupil reflex, GCS, and age	Tau			7.1, p < 0.01	5.7, p = 0.01
Pupil reflex, GCS, and age	Nfl			6.4, p = 0.01	6.1, p = 0.01
Pupil reflex, GCS, and age	Nfl	Tau		4.6, p = 0.03	3.3, p = 0.07

Results of Wald Chi-Squared Test of nested regression models analyzing the added value of Nfl and Tau for prediction of 1-year mortality and 6–12 months functional outcome in patients with TBI. Established clinical outcome predictors (GCS; Marshall score; and a model of pupil reflex score, GCS on admission, and age)) provided the basic models that Age, Nfl and Tau were added to in sequential manner. First, Tau and Nfl were included individually to each model. Then, Tau was included atop Nfl to test for further improvement of the model. A Chi-Square different from 0 with a p ≤ 0.05 represents a statistically significant improvement of the model by inclusion of the respective variable.

*Nfl* neurofilament light, *GCS* Glasgow coma scale.

## Discussion

This study confirms elevation of serum biomarker levels in patients with TBIs as well as the association between serum biomarkers and TBI severity in a cohort of unselected patients with multiple concomitant injuries. In this setting, our main finding is that addition of Nfl or T-Tau admission levels to commonly used clinical scores significantly improves prediction of outcome in patients with TBI. Moreover, addition of the combination of Nfl and T-Tau to predictive models improves the predictive value over that of a single biomarker. Lastly, the study reveals that specific biomarkers may be associated with admission injury characteristics (e.g., GFAP) whereas others mainly reflect prognosis (e.g., T-Tau).

Elevation of serum biomarkers in patients with traumatic brain and spinal cord injuries is well established but previous studies are small^29–31^, sample patients at significantly later time points after injury^18,19,32,33^ or use uninjured controls^8,18,19^. Confirming these prior findings in our unselected cohort of patients with multiple trauma and blood sampling very early after injury verify the clinical utility of the serum biomarkers in TBI and demonstrate their applicability in an acute setting^19,32,33^.

Several previous studies have reported an association between serum biomarkers and outcome after TBI^6,18,32,34^ but few evaluated their value alongside implemented clinical scores^35,36^. In our cohort, admission levels of T-Tau and Nfl were independently associated with outcome and improved the predictive value of GCS, Marshall, and an outcome model based on pupil reflex, GCS and age (alike the IMPACT model). This is in line with the recent CENTER-TBI study which reported improved prediction of the IMPACT model after addition of serum biomarkers^34^. Similar improvements of IMPACT scores have been reported also in smaller studies across different serum biomarkers, sampling times, and injury severities^32,37^. However, the IMPACT model is not implemented in clinical practice. Therefore, we extended the studies to more commonly used clinical scores and reproduced the additive value in combination with GCS and the Marshall score. This has been done in only few previous studies^6,36^, including Nimer et al. who reported additive value of Nfl to a model including core clinical scores in a cohort of 182 patients with mild to severe TBI^36^. As such, our results are in line with previous evidence and support the idea of applying biomarkers to established clinical scores to improve outcome prediction. It extends evidence by demonstrating biomarker applicability in the acute phase after injury.

In theory, a combination of biomarkers representing different cellular origins and pathological injury processes could provide a more comprehensive picture of TBI than a single biomarker. T-Tau atop Nfl improved outcome prediction by the majority of models. This is in line with the CENTER-TBI study which improved outcome prediction of the IMPACT model by addition of a panel of 6 serum biomarkers as opposed to a single biomarker^34^. In a smaller study, Thelin et al. improved outcome prediction by the IMPACT model by adding Nfl atop GFAP^32^, and Al Nimer et al. reported additive predictive value of Nfl to a model including S100B^36^. While not completely uniform, evidence suggests an additive value of serum biomarkers for outcome prediction after TBI, and our data support this view. Furthermore, it demonstrates that serum biomarker panels may have a readily applicable role in outcome prediction as an addition to established clinical outcome predictors.

Evidence is less clear as to which biomarkers to include in predictive models. We pursued the additive value of T-Tau and NfL because they were independently associated with outcome while GFAP and UCHL1 were only associated with outcome in crude analysis. In the CENTER-TBI study, predictive value was uncovered for all four biomarkers with the strongest associations identified for UCHL1 and T-Tau which led to the suggestion of combining UCHL1, Nfl, and T-Tau in a biomarker panel^34^. Earlier studies mainly presenting univariate analysis have observed predictive value of all four biomarkers^32^ or of GFAP and UCHL1 in patients with severe TBI^37,38^. However, individual biomarkers show distinct release patterns after TBI^32^, and release of UCHL1 may be associated with diffuse brain injury^38^ which was less prevalent in our cohort. This may explain why UCHL1 was not independently associated with outcome in our cohort and suggests that timing of blood sampling and the nature of brain injury are essential when selecting the best biomarker panel for outcome prediction.

Along this line, it is increasingly evident, that some serum biomarkers are mainly prognostic whereas others are better in diagnostics^34^. GFAP was strongly associated with presence of brain injury and increasing TBI severity in our study as in line with the literature^16,39–41^, including the ALERT-TBI^39^ and CENTER-TBI studies^34^. Conversely, T-Tau provided little diagnostic value but profoundly improved outcome prediction which was also observed in the CENTER-TBI study^34^. Nfl appears more versatile demonstrating both diagnostic and prognostic abilities in our as well as other studies^16,18^. Collectively, our study supports the biomarker panel suggested in the CENTER-TBI study for outcome prediction, also in acutely sampled patients. Furthermore, it highlights that specific serum biomarker panels are needed depending on intended clinical use.

This study is not without limitations. It was conducted on biobank material not collected specifically for this study^21^. However, the study design and cohort characteristics are well-suited for the present study. The TBI patients were significantly older than the controls which may have affected the results as the neurological biomarkers increase with age^42,43^. However, the expected increase per ten-year patient age is of limited magnitude compared with the responses observed following TBI^44^ and we adjusted all our analysis for age. As such, the impact of this imbalance is consider of limited importance. The grouping of patients was based on a retrospective chart review, and we cannot exclude the possibility that some control patients had unregistered TBI symptoms. However, this would reduce the difference between study groups leading to an underestimation of the difference in this study.

While it is among the largest TBI biomarker studies available, only a limited number of patients succumbed to their injuries (n = 21) or had an unfavorable functional outcome (n = 32). We therefore dichotomized functional outcome at a GOSE score of 4. While there is no standardized cut point for GOSE dichotomization this cut point is frequently used^34,45,46^.

We used an outcome model based on pupil reflex, GCS and age which approximates the IMPACT core model^4^ in our analysis. In this model GCS motor score was replaced by the total GCS score. This approximation is considered reasonable as total GCS and GCS motor score performs almost identical in prediction of mortality following TBI^47^.

The predictive models presented in this study lack cross-validation, due to the limited size of our cohort. With a larger sample size, our cohort would ideally have been divided into a training and a validation set to test for model overfitting. To reduce this risk, we limited each model to 3–4 predictive variables, as fit for our sample size.

Due to the limited number of TBI patients with worse outcomes, the association of serum biomarkers and outcome was assessed by several, simpler regression models which introduce a risk of type I error. However, our results are generally uniform over multiple models of the same outcome which makes type I errors less likely.

We analyzed serum biomarkers sampled upon hospital admission, to capture biomarker performance in an acute setting. By choosing this sampling time, we also limited the possible impact of in-hospital events on biomarker levels such as blood transfusions and surgical procedures.

## Conclusion

Our data confirm recent evidence of additive value of serum biomarkers for outcome prediction after TBI, also in the acute phase after injury. It demonstrates that serum biomarkers provide additive prognostic value to more commonly used outcome scoring systems and that a biomarker panel including Nfl and T-Tau improves outcome prediction in TBI further than each biomarker alone. Our results highlight the need for serum biomarker panels targeted at a specific clinical purpose. Collectively, this is an extension of the current knowledge that promotes the clinical applicability of serum biomarkers for outcome prediction in patients with TBI.

### Supplementary Information

Supplementary Tables.

## Data Availability

The data that support the findings of this study can be made available from the corresponding author, Claus V. B. Hviid, upon reasonable request.

## Abbreviations

AIS: Abbreviated injury severity score
ATLS: Advanced trauma life support
CNS: Central nervous system
CT: Computed tomography
CV: Coefficient of variation
IMPACT: International mission for prognosis and analysis of clinical trials in TBI
LLOD: Lower limit of detection
LLOQ: Lower limit of quantification
GCS: Glasgow coma scale
GOSE: Glasgow outcome score extended
GFAP: Glial fibrillary acidic protein
Nfl: Neurofilament light chain
NISS: New injury severity score
PRS: Pupil reflex score
T-Tau: Total-Tau
TBI: Traumatic brain injury
TSCI: Traumatic spinal cord injury
UCHL1: Ubiquitin C-terminal hydrolase L1
