# Supplementary material for: Admission levels of serum biomarkers have additive and cumulative prognostic value in traumatic brain injury
Source: Sci Rep. 2024 Jun 19;14:14139. doi: 10.1038/s41598-024-64125-1 (PMC11187066; doi:10.1038/s41598-024-64125-1)
Supplement: Supplementary file 1 — Supplementary Tables. [file 41598_2024_64125_MOESM1_ESM.docx]

**Supplementary material**

**Admission levels of serum biomarkers have additive and cumulative prognostic value in traumatic brain injury**

Ida A. Kaaber^1,2^; Maj Lesbo MD^3^; Thea O. Wichmann MD^4^; Dorte Aa. Olsen MSc^5^; Mikkel M. Rasmussen MD, PhD^2,4^; Ole Brink MD, PhD, MPA^6^; Lars C. Borris MD^6^; Claus V.B. Hviid MD, PhD^1,2,7,8^.

Table 1: Technical specifications of the analysis of serum biomarkers levels

Table 2: Handling of missing values

Table 3: Adjusted comparison of biomarker levels between TBI and control patient

Table 4: Biomarker levels in patients with reduced pupil reflex

Table 5: ROC curves for mortality and functional outcome

Table 6: Correlation between admission levels of serum biomarkers

**Supplementary table 1**

Title: Technical specifications of the analysis of serum biomarkers levels

Legend: Technical specifications of the analysis of serum biomarkers levels using the Neurology 4-plex assay B kit (Quanterix Corp, MA USA) on a Simoa HD-1 Analyzer (Quanterix Corp). Abbreviations: T-Tau: Total-Tau; Nfl: Neurofilament light; GFAP: Glial fibrillary acidic protein; UCHL1: Ubiquitin carboxy-terminal hydrolase L1; LLOQ: Lower limit of quantification; LOD: Lower limit of detection; CV: Coefficient of Variability; Simoa: Single Molecule Array.

| Serum biomarker | **T-Tau** | **Nfl** | **GFAP** | **UCHL1** |
| --- | --- | --- | --- | --- |
| LLOQ, pg/ml | 0.125 | 0.500 | 9.38 | 9.38 |
| LOD, pg/ml | 0.0408 | 0.105 | 1.51 | 1.90 |
| Calibrator range at 1:4 dilution, pg/ml | 0-400 | 0-2000 | 0-40000 | 0-40000 |
| CV, %  Sample level | 10.1%  2.4 pg/ml | 13.1 %  5.8 pg/ml | 11.0 %  128.73 pg/ml | 10.7 %  115.12 pg/ml |
| CV for high control sample, % | 9.5%  81.3 pg/ml | 18.6%  532.0 pg/ml | 14.0%  11711.1 pg/ml | 9.0%  8887.3 pg/ml |

**Supplementary table 2**

Handling of missing values

The “time from injury to first sample” was median 72 mins (IQR: 50 to 95) and mean 153 ± 267 mins when the substituted values were included. This decreased to median 69 mins (IQR: 50 to 90) and mean 71 ± 26 mins after exclusion of these values.

The median biomarker levels (IQR) in the first sample is outlined in the table below:

|  | Substitution performed | Substitution not performed |
| --- | --- | --- |
| Tau | 6.8 (3.1 to 16.8), N=236 | 6.9 (3.3 to 16.9), N=214 |
| NfL | 12.2 (6.8 to 28.4), N=236 | 11.1 (6.6 to 24.9), N=214 |
| GFAP | 500.3 (164.7 to 2728.3), N=236 | 439.10 (155.6 to 1678.2), N=214 |
| UCHL1 | 73.7 (35.7 to 162.8), N=233 | 76.3 (36.1 to 163.6), N=212 |

Lastly, we evaluated the correlation between time to first sample with biomarker level in the collective cohort by Spearmans Rho. These results are presented below:

|  | Spearmans Rho | p-value |
| --- | --- | --- |
| T-Tau | 0.07 | 0.237 |
| NfL | 0.211 | <0.001 |
| GFAP | 0.293 | <0.001 |
| UCHL1 | 0.167 | <0.01 |

| **Supplementary 3** Association between serum biomarker levels on admission, at 15, and 72 hours and TBI status | | | | | | | | |
| --- | --- | --- | --- | --- | --- | --- | --- | --- |
|  | **Tau** | | **Nfl** | | **GFAP** | | **UCHL1** | |
|  | Crude | Adjusted | Crude | Adjusted | Crude | Adjusted | Crude | Adjusted |
| Admission | | | | | | | | |
| TBI | 1.44 (1.07 to 1.95),  p=0.02 | 1.16 (0.90 to 1.50),  p=0.25 | 1.53 (1.25 to 1.80),  p<0.001 | 1.16 (1.01 to 1.34),  p=0.04 | 4.68 (3.36 to 6.52),  p<0.001 | 3.32 (2.51 to 4.39),  p<0.001 | 1.83 (1.37 to 2.45),  p<0.001 | 1.50 (1.16 to 1.95),  p<0.01 |
| 15 hours | | | | | | | | |
| TBI | 1.72 (1.24 to 2.37),  P=0.001 | 1.60 (1.18 to 2.17),  p<0.01 | 2.24 (1.69 to 2.95),  p<0.001 | 1.81 (1.45 to 2.27),  p<0.001 | 11.87 (7.48 to 18.83),  p<0.001 | 9.78 (6.43 to 18.88),  p<0.001 | 1.94 (1.32 to 2.85),  p=0.001 | 1.72 (1.21 to 2.44),  p<0.01 |
| 72 hours | | | | | | | | |
| TBI | 2.02 (1.32 to 3.08),  p=0.001 | 1.69 (1.10 to 2.59),  p=0.02 | 2.65 (1.82 to 3.84),  p<0.001 | 2.07 (1.46 to 2.94),  p<0.001 | 15.72 (8.55 to 28.91),  p<0.001 | 11.24 (6.15 to 20.56),  p<0.001 | 2.75 (1.69 to 4.46),  p<0.001 | 2.52 (1.51 to 4.19),  p=0.001 |

**Supplementary 3** Results of multiple linear regression analyses for the association between serum biomarker levels on admission, 15-, and 72- hours with TBI group status. The β coefficients were back-transformed to the original scale and are presented with 95% confidence intervals. Adjustments for age, sex, and NISS. Abbreviations: Nfl: Neurofilament light; GFAP: Glial fibrillary acidic protein; UCHL1: Ubiquitin carboxy-terminal hydrolase L1; NISS; New Injury Severity Scale.

**Supplementary table 4.**

Biomarker levels in TBI patients without/with pupil reflex. Biomarker levels presented as median with with interquartile range. P-values for comparison between +/- pupil reflex groups presented. Abbreviations T-Tau: total-tau; Nfl: Neurofilament light; GFAP: Glial fibrillary acidic protein; UCHL1: Ubiquitin carboxy-terminal hydrolase L1

|  |  | + pupil reflex | - pupil reflex |  |
| --- | --- | --- | --- | --- |
| Tau | Adm | 6.7 (2.9 to 15.7)  N=222 | 19.6 (4.2 to 672.4)  N=14 | p<0.01 |
|  | 15 | 3.6 (2.2 to 7.0)  N=154 | 83.9 (2.8 to 215.8)  N=11 | p<0.01 |
|  | 72 | 2.6 (1.2 to 4.6)  N=70 | 24.6 (5.3 to 63.6)  N=9 | p<0.001 |
| NfL | Adm | 11.3 (6.6 to 24.4)  N=222 | 43.3 (29.6 to 148.0)  N=14 | p<0.001 |
|  | 15 | 23.8 (12.8 to 51.9)  N=154 | 221.0 (27.5 to 529.8)  N=11 | p<0.01 |
|  | 72 | 45.6 (27.1 to 93.1)  N=70 | 142.9 (65.9 to 462.5)  N=9 | p<0.01 |
|  | Adm | 439.0 (156.2 to 2033.6)  N=222 | 5732.4 (1678.2 to 46818.2)  N=14 | p<0.001 |
| GFAP | 15 | 4255.1 (736.9. to 12135.3)  N=154 | 4967.9 (1925.6 to 47675.8)  N=11 | p=0.10 |
|  | 72 | 1907.1 (307.3 to 8449.6)  N=70 | 6371.8 (4208.1 to 29940.5)  N=9 | p=0.01 |
| UCHL1 | Adm | 71.7 (34.4 to 155.7)  N=219 | 117.9 (74.0. to 2554.1)  N=14 | 0.03 |
|  | 15 | 50.7 (25.7 to 126.4)  N=146 | 270.8 (55.2 to 639.2)  N=11 | <0.01 |
|  | 72 | 33.9 (15.2 to 67.6)  N=65 | 70.9 (27.6 to 132.2)  N=9 | 0.09 |

**Supplementary table 5**

| **Supplementary table 5** Comparisons of ROC curves of admission levels of serum biomarkers for separating TBI patients by 1-year mortality and 6-12 months functional outcome | | |
| --- | --- | --- |
| **1-year mortality** | | |
| Nfl vs. Tau | 0.88 (0.82 to 0.94) vs. 0.78 (0.67 vs. 0.89) | p=0.07 |
| Nfl vs. GFAP | 0.88 (0.82 to 0.94) vs. 0.80 (0.69 to 0.91) | p=0.03 |
| Nfl vs. UCHL1 | 0.88 (0.82 to 0.94) vs. 0.74 (0.63 to 0.86) | p<0.01 |
| Tau vs. GFAP | 0.78 (0.67 vs. 0.89) vs. 0.80 (0.69 to 0.91) | p=0.75 |
| Tau vs. UCHL1 | 0.78 (0.67 vs. 0.89) vs. 0.74 (0.63 to 0.86) | p=0.46 |
| GFAP vs. UCHL1 | 0.80 (0.69 to 0.91) vs. 0.74 (0.63 to 0.86) | p=0.35 |
| **6-12 months functional outcome** | | |
| Nfl vs. Tau | 0.84 (0.77 to 0.90) vs. 0.75 (0.65 to 0.85) | p=0.16 |
| Nfl vs. GFAP | 0.84 (0.77 to 0.90) vs. 0.84 (0.77 and 0.91) | p=0.91 |
| Nfl vs. UCHL1 | 0.84 (0.77 to 0.90) vs. 0.76 (0.67 to 0.85) | p=0.15 |
| Tau vs. GFAP | 0.75 (0.65 to 0.85) vs. 0.84 (0.77 and 0.91) | p=0.11 |
| Tau vs. UCHL1 | 0.75 (0.65 to 0.85) vs. 0.76 (0.67 to 0.85) | p=0.73 |
| GFAP vs. UCHL1 | 0.84 (0.77 and 0.91) vs. 0.76 (0.67 to 0.85) | p=0.12 |

**Supplementary table 5** Comparisons of ROC curves of admission levels of serum biomarkers for separating TBI patients by 1-year mortality and 6-12 months functional outcome. AUCs (95%-CI) of each biomarker model and p values of the test of equality presented. Abbreviations: ROC: Receiver operating characteristics; Nfl: Neurofilament light; GFAP: Glial fibrillary acidic protein; UCHL1: Ubiquitin carboxy-terminal hydrolase L1; NISS; New Injury Severity Scale.

| **Supplementary table 6** Correlation between admission levels of serum biomarkers | | |
| --- | --- | --- |
| Tau/Nfl | ρ=0.33 | p<0.001 |
| Tau/GFAP | ρ=0.40 | p<0.001 |
| Tau/UCHL1 | ρ=0.62 | p<0.001 |
| Nfl/GFAP | ρ=0.61 | p<0.001 |
| Nfl/UCHL1 | ρ=0.45 | p<0.001 |
| GFAP/UCHL1 | ρ=0.61 | p<0.001 |

**Supplementary table 6** Correlation between admission levels of serum biomarkers assessed with Spearman’s rho (ρ). Abbreviations: Nfl: Neurofilament light; GFAP: Glial fibrillary acidic protein; UCHL1: Ubiquitin carboxy-terminal hydrolase L1.
